# Exosome in renal cell carcinoma progression and implications for targeted therapy

**DOI:** 10.3389/fonc.2024.1458616

**Published:** 2024-09-04

**Authors:** Xinwei Li, Wen Xiao, Hongmei Yang, Xiaoping Zhang

**Affiliations:** ^1^ Department of Urology, Union Hospital, Tongji Medical College, Huazhong University of Science and Technology, Wuhan, China; ^2^ Institute of Urology, Union Hospital, Tongji Medical College, Huazhong University of Science and Technology, Wuhan, China; ^3^ Department of Pathogenic Biology, School of Basic Medicine, Huazhong University of Science and Technology, Wuhan, China; ^4^ Shenzhen Huazhong University of Science and Technology Research Institute, Shenzhen, China

**Keywords:** exosome, extracellular vesicles, renal cell carcinoma, tumor progression, targeted therapy

## Abstract

Renal cell carcinoma is a urological malignancy with a high metastatic rate, while targeted therapy for renal cell carcinoma still has much room for improvement. Some cutting-edge researches have focused on exosome in cancer treatment and there are some breakthroughs in breast cancer, lung cancer, and pancreatic cancer. Up to now, exosome in renal cell carcinoma progression and implications for targeted therapy has been under research by scientists. In this review, we have summarized the structure, formation, uptake, functions, and detection of exosomes, classified the mechanisms of exosomes that cause renal cell carcinoma progression, and listed the promising utilization of exosomes in targeted therapy for renal cell carcinoma. In all, based on the mechanisms of exosomes causing renal cell carcinoma progression and borrowing the successful experience from renal cell carcinoma models and other cancers, exosomes will possibly be a promising target for therapy in renal cell carcinoma in the foreseeable future.

## 1 Introduction

Renal cell carcinoma (RCC) is a urological malignancy with an increasing incidence in recent years, which accounts for about 3% of malignancies in adults (1, 2). In 2022, about 79,000 new cases and 13,920 deaths were reported in the United States, while about 77,410 new cases and 46,345 deaths were reported in China (3). According to pathological examinations, RCC could be classified as clear cell renal cell carcinoma (ccRCC), papillary RCC, chromophobe RCC, translocation-associated RCC, medullar RCC, and collecting duct RCC, while ccRCC is the most common pathological type, which takes up about 75% of RCC (4). RCC incidence rises with age and is more prevalent in men than women, with major risk factors including excess body weight, hypertension, and cigarette smoking. For ccRCC, it is characterized by frequent mutations in the VHL tumor suppressor gene, leading to the activation of genes related to angiogenesis, glycolysis, and apoptosis. Besides, additional genetic and epigenetic alterations are required for ccRCC development. The tumor microenvironment of ccRCC is characterized by high T cell infiltration, particularly in higher-grade and stage tumors, with increased T helper 2 and T regulatory cell infiltration (5). RCC often presents without symptoms in its early stages, with many cases being incidentally discovered during imaging studies performed for unrelated reasons. When symptoms do appear, they can include flank pain, hematuria, and a palpable abdominal mass. The diagnosis of RCC relies on computed tomography (CT) or magnetic resonance imaging (MRI) to assess the size, location, and extent of the tumor (6). Though around 70% of patients were diagnosed with localized RCC and complete nephrectomy was performed after diagnosis, 30% of them would develop metastasis in the follow-up, whereas around 30% of patients were diagnosed with metastatic RCC in the beginning (4, 7). The drugs for metastatic RCC have improved a lot in recent years, which mainly include targeted therapy (TT) agents and immune checkpoint inhibitors (ICIs). The former could be subdivided into inhibitors of vascular endothelial growth factor (VEGF) signaling such as sunitinib and inhibitors of mammalian target of rapamycin (mTOR) including temsirolimus, and the latter contains programmed death-1 (PD-1), programmed death ligand-1 (PD-L1), and the cytotoxic T-lymphocyte associated protein-4 (CTLA-4) (8). Nonetheless, with the combination of targeted therapy and immunotherapy for metastatic RCC, the average survival time is only 48 months, and drug resistance and immune-related adverse events (irAEs) are also unsolved problems (9).

Recently, more and more researches have cast light on exosomes in cancer treatment including breast cancer, lung cancer, and pancreatic cancer, and the relationship between tumor metastasis and exosome is a cutting-edge topic (10, 11). In therapy for breast cancer, exosomes are modified with active cancer-targeting folic acid (FA) on the surface to carry indocyanine green (ICG) towards the cancer cells, which could accumulate in tumor cells and suppress the tumor growth significantly (12). As for lung cancer, some scientists have constructed exosomes that could be the carriers for siRNA-loaded PD-L1, which could target lung cancer cells (13). Moreover, depending on CD47, the modified exosomes could target oncogenic Kirsten rat sarcoma viral oncogene homolog (KRAS) via micropinocytosis, which would suppress pancreatic cancer progression and increase the survival rate in mice models (14). Given their ability to carry molecular cargo, it is not surprising that exosomes are emerging as a promising target for RCC treatment. Many medical professionals are optimistic that these novel targeted therapies will be effective against this challenging malignancy. To date, most of the reviews about exosome and RCC on the Internet have only focused on the diagnosis of RCC through exosomal molecules and the mechanisms of RCC drug resistance caused by exosomes, while there is no review about the mechanisms that exosome causes RCC progression and exosome as a promising new target for RCC treatment. In this review, we will discuss the mechanisms of exosome-mediated RCC progression and find out potential ways for targeted therapies to inhibit RCC progression. Possibly, based on the mechanisms of exosomes causing renal cell carcinoma progression and borrowing the successful experience from renal cell carcinoma models and other cancers, exosomes will possibly be a promising target for therapy in renal cell carcinoma in the foreseeable future.

## 2 Structure, formation, uptake, and functions of exosome

**Figure 1:**
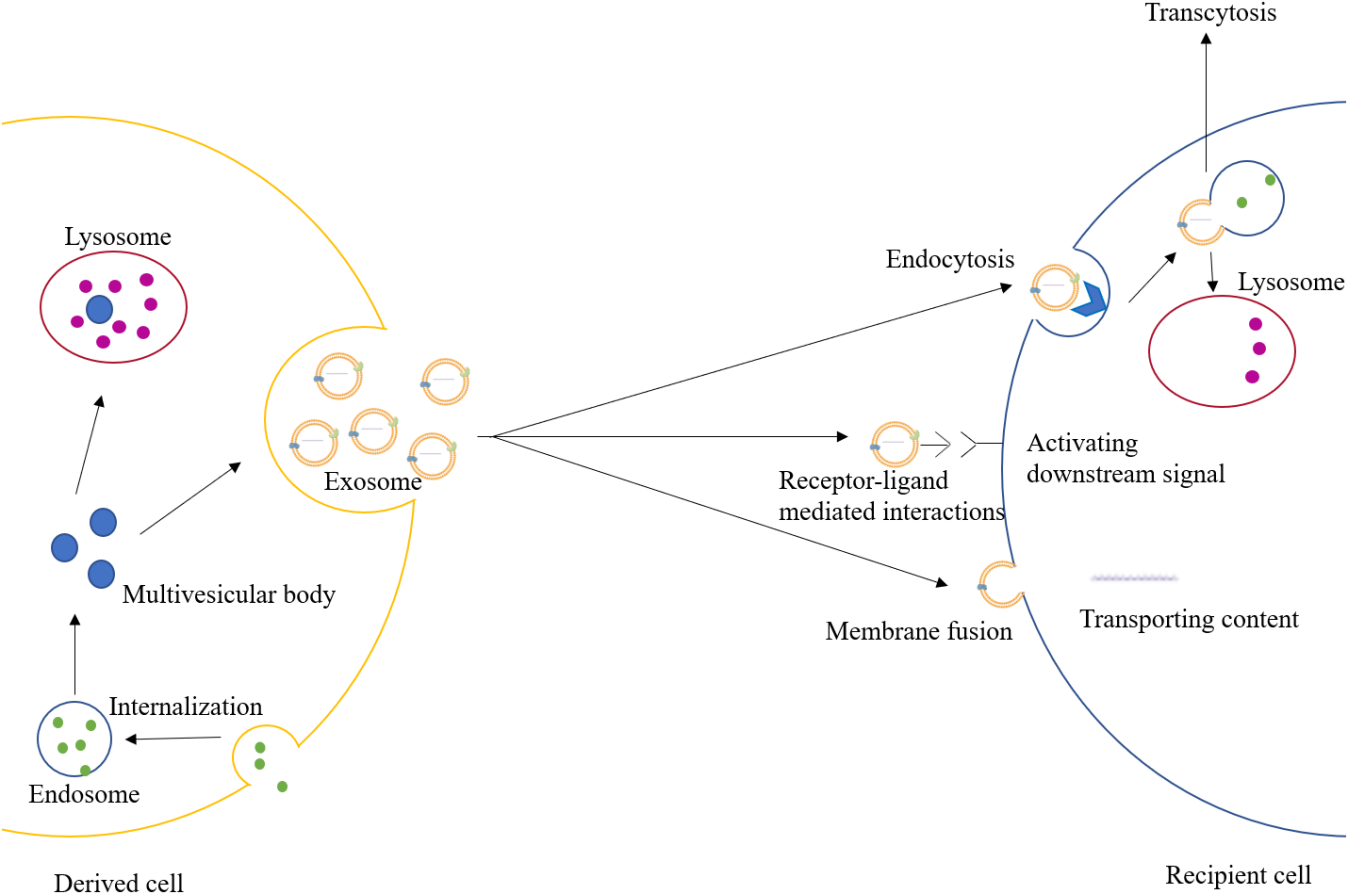
The formation and uptake of exosomes.

Extracellular vesicles are a class of cell-derived membrane structures consisting of exosomes and microvesicles that originate from the endosomal system or are shed from the plasma membrane, respectively (15). Exosome, a membrane-bound vesicle of 30-150 nm in diameter and also known as intraluminal vesicle (ILV), can be secreted by all kinds of cells and has been found in plasma, serum, lymph, urine, semen, saliva, bile, gastric acid, etc. (16, 17). The membrane of the exosome mainly consists of lipid and protein, which is rich in lipid raft, and multiple kinds of molecules have been found in the lumen of the exosome (18). Exosomes contain specific membrane proteins, lipids, nucleic acids, cell membrane proteins, and other signaling molecules. These molecules can be trafficked to recipient cells by exosomes, and the mechanisms by which different molecules select carriers vary (19) (**Table 1**). The formation of the exosome starts with the formation of the endosome, which is produced by the internalization of the cell membrane. Later, the endosome would be separated into many small vesicles named multivesicular bodies (MVBs). Some of the MVBs would be degraded by lysosomes or autophagosomes, while others would fuse with the cell membrane and release the ILVs to the extracellular region as exosomes (18, 20). After being secreted, the exosome could exchange information through endocrine, paracrine, and autocrine. When the exosome reaches the recipient cells, three mechanisms can explain its uptake: receptor-ligand mediated interactions, membrane fusion, and endocytosis (21). The receptor-ligand mediated interactions mainly activate the downstream signal in the recipient cell through signal transduction. The membrane fusion relies on two distinct membranes getting close and forming a fusion pore, which could transport the contents in the exosomes into the cytoplasm (22). The endocytosis process would require various transmembrane receptors to form coated vesicles for internalization, and later the vesicles would be uncoated and fuse with endosomes, which would undergo transcytosis and would be released towards the neighboring cells through paracrine, or they would be degraded by lysosomes (18, 23). The degradation products in the lysosomes and the contents trafficked through membrane fusion could also activate physiological and pathological responses (21). (**Figure 1**)

**Table 1:** Mechanisms of molecules selecting carriers.

Biological Molecules	Main Components	Sorting Mechanisms
Proteins	Transmembrane proteins (e.g., tetraspanins), membrane-associated proteins (e.g., flotillins), and soluble proteins	Interactions with ESCRT complexes Plasma membrane budding/shedding
RNA	mRNA, miRNAs, snRNAs, circRNAs, lncRNAs, etc.	Active loading through specific motifs (EXOmotifs) and RBPs (e.g., hnRNPA2B1, FMRP) Passive loading depending on intracellular RNA concentration
DNA	Genomic dsDNA, dsDNA-binding histones, ssDNA, and mtDNA	Mechanisms not fully elucidated (may involve cytoprotective functions)
Lipids	Cholesterol, phosphatidylcholine, phosphatidylserine, sphingomyelin, and ceramide	Various ESCRT-independent mechanisms

The exosome mainly functions as a carrier for the circulation of lipids, proteins, and nucleic acids in the extracellular region, which may be important for intercellular communication, immune response, tumor progression, etc. (16, 18). For instance, the communications between intestinal epithelial cells (IECs) and dendritic cells (DCs) rely on exosome loading with peptide-MHC II complexes, which play a significant role in inflammatory bowel diseases (IBDs) (24). Besides, Hamzah et al. have claimed that the uptakes of different types of exosomes by neuronal cells have different effects, though the mechanisms behind them are still unknown (25). Additionally, Lu and colleagues have proved that exosomes produced by senescent osteoblasts could upregulate the miR-139-5p in endothelial cells (26). In neurodegenerative diseases, the expression of exosomal miRNAs could be influenced by oxidative stress, while the exosomal miRNAs could also affect the oxidative stress response through gene regulation (27). Additionally, Noonin et al. summarized the crosstalk between exosomes and inflammasomes. For one thing, the release of exosome could be regulated by the inflammasome, for another exosome could also have an impact on the activation of inflammasome, while whether exosome is alleviating or enhancing inflammasome activation depends on what kind of cells are producing exosomes (28). The opinion that exosomes played a key role in the pathological process of diabetes was proposed by Chang et al. On the one hand, exosomes connect the adipocyte stimulation with insulin resistance and the immune cell response with pancreatic tissue injury. On the other hand, the nucleic acids in the exosomes, especially miRNA and lncRNA, could regulate the communications between organs involved in the pathological processes of diabetes, such as impacting the metabolic signals and insulin signals in the targeted tissues (29). Furthermore, exosomes could also traffic biologically functional molecules towards the recipient cells, which could reprogram the tumor cells and modulate the metabolism of the stromal cells in the tumor microenvironment (TME) (30). Exosomes promote angiogenesis by carrying pro-angiogenic factors like VEGF to stimulate the formation of new blood vessels, supporting tumor growth and metastasis. In terms of immune modulation, exosomes can carry immunosuppressive molecules like PD-L1, inhibiting the immune cells to attack the tumor. Exosomes affect cancer stem cells by regulating their self-renewal and differentiation, contributing to the maintenance of cancer stem cell populations within the tumor. Additionally, they can transfer drug resistance mechanisms between cancer cells helping them resist chemotherapy. This would promote the progression, metastasis, and drug resistance of the tumor (31).

## 3 Detection of exosomes

The process of detecting exosomes is usually divided into several steps: isolation, purification, identification, and analysis. Traditional methods of isolating and purifying exosomes include ultracentrifugation, ultrafiltration, size-exclusion chromatography, immunoaffinity, and polymer precipitation, while more recent methods include membrane based-separation and microfluidic technology (32). The identification and analysis mainly rely on nanoparticle tracking analysis, dynamic light scattering, resistive pulse sensing, atomic force microscopy, transmission electron microscopy, flow cytometry, while new approaches such as filter paper-based techniques, electrochemical sensing, optical approaches present low sample volumes and potentially much lower operational costs (33).

## 4 The mechanisms of exosome cause RCC progression

Tumor metastasis can’t be separated from intercellular communication. Before the metastasis occurs, not only would tumor cells transduce signals to the primary tumor microenvironment, but tumors would also send signals towards the expected sites for future metastasis, which could form a hospitable pre-metastatic niche (PMN) for the arrival of tumor cells later (34). In detail, exosomes could regulate the permeability of local vessels to make tumor progression more convenient. Besides, to achieve the goal of sending signals to metastatic sites, exosomes act as carriers for nucleic acids and proteins. They regulate the physiology of distant non-tumor cells, making potential metastatic sites suitable for the dissemination and growth of metastatic tumor cells (35). Interestingly, the destinations of tumor metastasis are decided by the exosomal integrin expressions. For instance, Hoshino et al. reported that integrins α6β4 and α6β1 were related to lung metastasis and exosomal integrin αvβ5 had a close connection with liver metastasis (36). Besides, some papers have also proved that the exosomes could also be trafficked from the cells in the TME to tumor cells, or even from one tumor cell to another tumor cell, which will be described in detail in the following parts (**Table 2**).

**Table 2:** Molecules conveyed by exosomes in the metastasis of RCC.

Conveyance direction	Contents in exosome	Function
RCC to TME	lncARSR	Inducing the M2 macrophage polarization.
	circSAFB2	Inducing the M2 macrophage polarization.
	AP000439.2	Promoting RCC metastasis and inducing M2 macrophage polarization.
	TGF-β1	Leading to the dysfunction of NK cells.
	ApoC1	Enhancing RCC metastasis.
TME to RCC	miR-19b-3p	Promoting RCC metastasis and epithelial-mesenchymal transition.
	miR-193a-5p	Increasing vasculogenic mimicry and tumor progression.
	Not mentioned	Enhancing RCC progression and suppressing RCC apoptosis.
	miR-224-5p	Modulating RCC progression, metastasis, invasion, and apoptosis.
	miR-181d-5p	Promoting RCC stemness and inducing metastasis.
	miR-155-5p	Promoting RCC proliferation and progression.
	miR-21-5p	Regulating RCC metastasis.
	miR-342-3p	Promoting RCC proliferation and invasion.
RCC to RCC	circ-PRKCI	Promoting RCC proliferation and progression.
	miR-15a	Facilitating tumor progression.
	lncHILAR	Inducing RCC metastasis.

### 4.1 Transportation of exosomes from RCC cells to other cells to regulate TME

**Figure 2:**
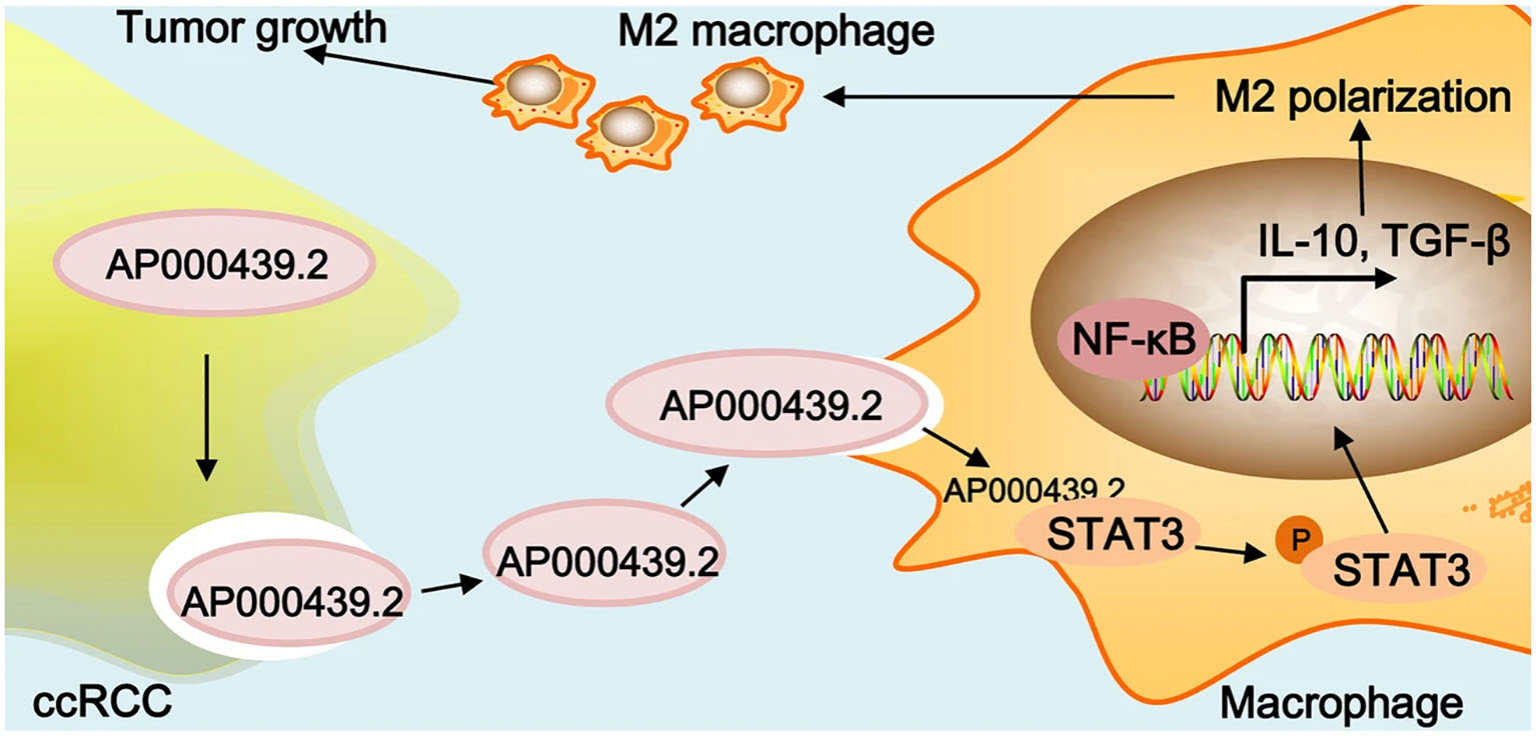
Graphical summary of lncRNA AP000439.2 regulation. Reprinted with permission from Shen T, Miao S, Zhou Y, Yi X, Xue S, Du B, et al. Exosomal AP000439.2 from clear cell renal cell carcinoma induces M2 macrophage polarization to promote tumor progression through activation of STAT3. Cell communication and signaling: CCS. 2022;20(1):152. Copyright Springer Nature.

In the progression of RCC, exosomes could function as the carriers to transfer small molecules from RCC cells to their destination to make the TME more suitable for tumor metastasis. Zhang et al. have explored that the RCC-derived exosomes contain a large number of lncRNAs named lncARSR. The overexpression of lncARSR could not only regulate the phenotype transformation and function of macrophages but also promote the RCC progression. The lncARSR could also interact with miR-34/miR-449 to upregulate the expression of signal transducer and activator of transcription 3 (STAT3) to induce the polarization of M2 macrophages in RCC, which may lead to tumor progression and metastasis due to immunosuppression function of M2 macrophages (37). Similarly, another research team has shown that RCC-derived exosomes with high expression of circSAFB2 can induce the polarization of M2 macrophages through the miR-620/JAK1/STAT3 pathway (38). A similar idea was also claimed by Shen et al. that AP000439.2 translated from lncRNA AP000439.2 in the ccRCC-derived exosomes could interact with STAT3 proteins and phosphorylate STAT3 in macrophages, which would lead to the overexpression of AP000439.2. In this way, the overexpression of AP000439.2 could also increase the p65 phosphorylation and the expression of TGF-β and IL-10, which would cause M2 macrophage polarization and tumor progression and metastasis (39) (**Figure 2**). Furthermore, exosomes derived from ccRCC could activate the TGF-β/SMAD signaling pathway, resulting in the dysfunction of NK cells and evasion of tumor immune surveillance (40). In addition, Li et al. have found that the ApoC1 in the ccRCC-derived exosome could be trafficked to vascular endothelial cells to promote metastasis through activating STAT3. STAT3 is also a transcriptional regulator of VEGF, which promotes neovascularization. This contributes to tumor growth and spread by providing oxygen and nutrients to the tumor (41)..

### 4.2 Transportation of exosomes from TME to RCC cells to induce the proliferation and metastasis of RCC cells

**Figure 3:**
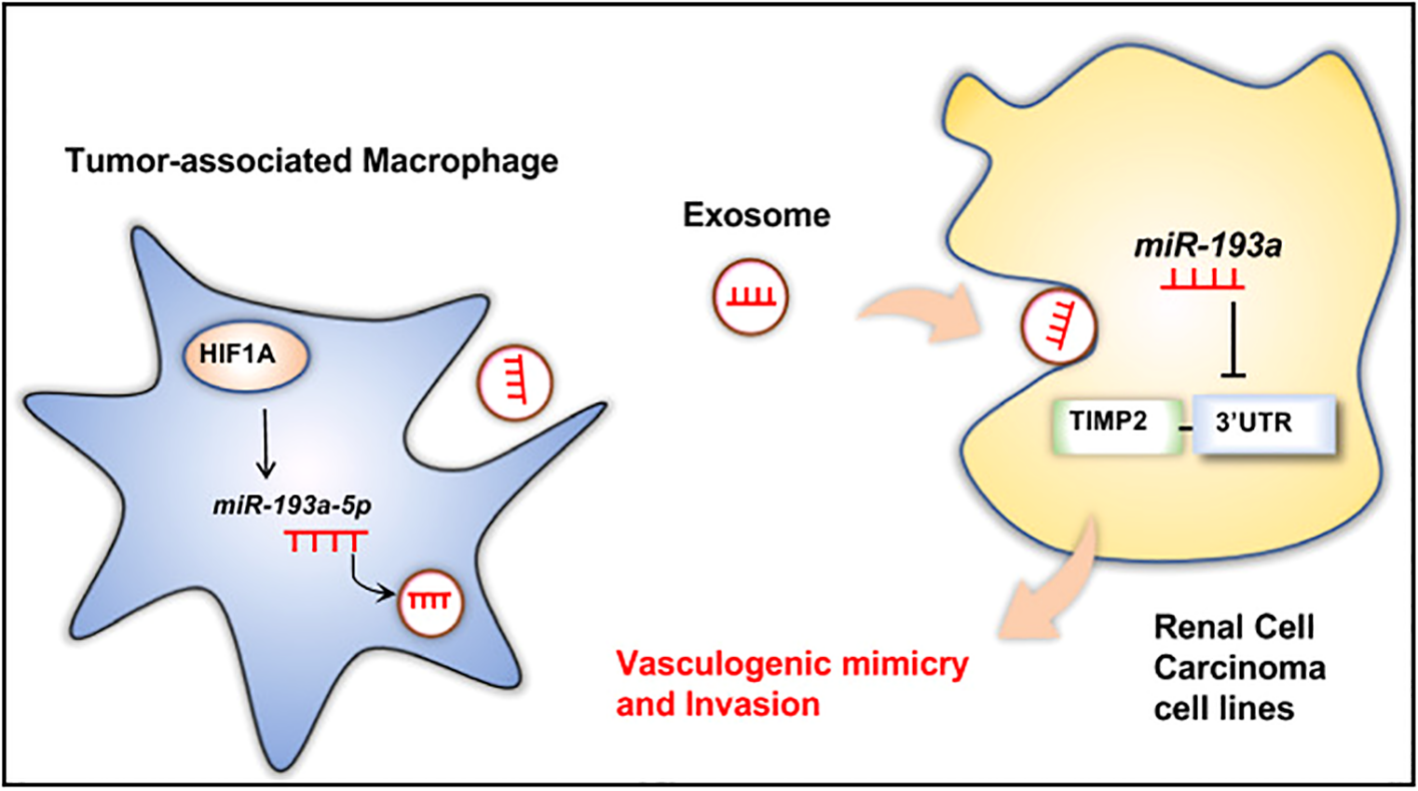
TAM-derived exosomal miR-193a-5p downregulates TIMP2 expression to facilitate vasculogenic mimicry and invasion of RCC cells. Reprinted with permission from Liu Q, Zhao E, Geng B, Gao S, Yu H, He X, et al. Tumor-associated macrophage-derived exosomes transmitting miR-193a-5p promote the progression of renal cell carcinoma via TIMP2-dependent vasculogenic mimicry. Cell death & disease. 2022;13(4):382. Copyright Springer Nature.

Besides, exosomes could also be transferred from the cells in the TME to RCC cells to induce tumor proliferation and metastasis. Wang et al. have claimed that exosomes derived from cancer stem cells (CSCs) could deliver miR-19b-3p towards ccRCC cells, which could promote tumor metastasis and the epithelial-mesenchymal transition (EMT). What was worth mentioning was that CD103+ CSC-derived exosomes could function as the guide for lung metastasis (42). Besides, the upregulation of HIF-1α in macrophages could increase the expression of miR-193a-5p, and later the exosomes produced by tumor-associated macrophages (TAMs) could traffic the miR-193a-5p towards the 3’ untranslated region (UTR) of TIMP2 mRNA in the RCC cells. The translation of TIMP2 mRNA would be inhibited, resulting in the increase of vasculogenic mimicry (VM) and tumor progression (43) (**Figure 3**). Moreover, exosomes produced by cancer-associated fibroblasts (CAFs) could be internalized by RCC cells, resulting in the enhancement of the proliferation, progression, and migration of the tumor cells, while the tumor cell apoptosis would be suppressed. Besides, the exosomes could also increase the proportion of S phase cells and the expression of fibronectin, N-cadherin, vimentin, MMP9, and MMP2 in tumor cells (44). Also, Liu et al. gave an example that exosomes secreted by CAFs would act as a carrier for miR-224-5p, which could be internalized by ccRCC cells to modulate the progression, metastasis, invasion, and apoptosis (45). Similarly, researchers have also reported that the miR-181d-5p in the exosomes could trafficked from CAFs to RCC cells, which could activate the Wnt/β-catenin signaling pathway to promote cancer stemness and tumor progression. In addition, ring finger protein 43 (RNF43), a direct target of miR-181d-5p, was a negative regulator of the Wnt/β-catenin signaling pathway. The miR-181d-5p could also suppress the expression of RNF43 to induce RCC metastasis (46). In addition, extracellular vesicles derived from CAFs transfer SNHG1 to RCC cells, leading to increased expression of SNHG1 in RCC cells. The exosomes secreted by CAFs promote the proliferation, migration, and invasion of RCC cells, while SNHG1 knockdown weakens the promoting effect of CAFs exosomes on RCC progression (47). Furthermore, exosomes produced by hypoxic TAMs could transport miR-155-5p to RCC cells, which could promote the progression and metastasis of the tumor cells via the IGF1R/PI3K/AKT pathway. Besides, the miR-155-5p could also increase the stability of IGF1R mRNA by interacting with human antigen R (HuR) to promote the proliferation and progression of RCC (48). Additionally, Zhang et al. have explored that exosomes produced by the pro-tumorigenic M2 macrophages could transport miR-21-5p to RCC cells. The miR-21-5p would target PTEN-3’UTR to regulate the PTEN/Akt signaling pathway, resulting in RCC metastasis (49). Similarly, another study has proved that M2 macrophage-derived extracellular vesicles could highly express miR-342-3p and transport it to RCC cells, which would specifically bind NEDD4L and suppress the expression of NEDD4L. This could improve the expression level of the CEP55 protein, promoting RCC proliferation and invasion through the PI3K/AKT/mTOR pathway (50).

### 4.3 Transportation of exosomes from RCC cells to RCC cells to promote metastasis

Apart from the communications via exosomes between RCC cells and TME, there also exists communications between RCC cells to promote tumor metastasis. For example, Qian et al. found that the exosomes derived from the RCC could deliver circ-PRKCI from highly malignant tumors to comparatively less malignant tumors, which could promote RCC proliferation and progression. In detail, the circ-PRKCI could promote RCC progression through the miR-545-3p/CCND1 pathway (51). Besides, other doctors have explored that miR-15a in the exosome produced by the RCC cells could activate the PI3K/AKT signaling pathway, which could lead to RCC proliferation, invasion, metastasis, and EMT. Moreover, B-cell translocation gene 2 (BTG2), a target gene of miR-15a, was negatively correlated with miR-15a expression and could inhibit the RCC proliferation. The miR-15a could also downregulate the expression of BTG2 and facilitate tumor progression (52). In addition, Hu and colleagues have explored the impact of hypoxia on RCC metastasis. The exosomes produced by hypoxic RCC cells could carry lncHILAR towards normoxic RCC cells. The lncHILAR would function as a competing endogenous RNA (ceRNA) for miR-613/206/1-1-3p, resulting in the upregulation of Jagged-1 and the C-X-C motif chemokine receptor 4 (CXCR4). After being activated, Jagged-1/Notch/CXCR4 axis would induce RCC metastasis (53). Another interesting article found that exosomes produced by VHL (–) RCC cells can induce EMT, migration, invasion, and distant metastasis of VHL (+) RCC cells after being uptaken (54).

## 5 Promising utilization of exosome in targeted therapy for RCC

Based on the function of intercellular transportations, exosomes could traffic small molecules to specific cells or tissues, which may play an important role in targeted therapy (55). On the one hand, exosomes could effectively prevent nucleic acid drugs from being degraded or neutralized by intracellular enzymes when certain drugs have to be transported into the target cells (56). On the other hand, to prevent exosomes from promoting tumor metastasis via intercellular communication, targeted therapies could aim at blocking exosome generation, secretion, and uptake (57). Up to now, exosomes have been applied to treating various diseases including infectious diseases, cardiovascular diseases, and nervous system diseases (58). A review published by Shi et al. has summarized that endogenous exosomes may be a promising tool to carry the functional Cas9 and HBV-specific gRNA to cut HBV DNA in HBV DNA transfected cells (59). Besides, Bu et al. have developed a system that could modify IL-10 mRNA with two miR-155 recognition sites. When the miR-155 in the macrophages was expressed, the IL-10 mRNA in the exosome could be delivered into macrophages and other cells in the plaque, and later the IL-10 mRNA would translate into protein, which could alleviate the atherosclerosis in the model (60). Another team has reported a device that could design the exosome to enhance exosome production, specific mRNA packaging, and delivery of the mRNA into the cytosol of target cells. This was used in the model for trafficking mRNA to the brain, which could alleviate neurotoxicity and neuroinflammation in models of Parkinson’s disease (61).

### 5.1 Utilizing the intercellular communications of exosomes to inhibit RCC metastasis

**Figure 4:**
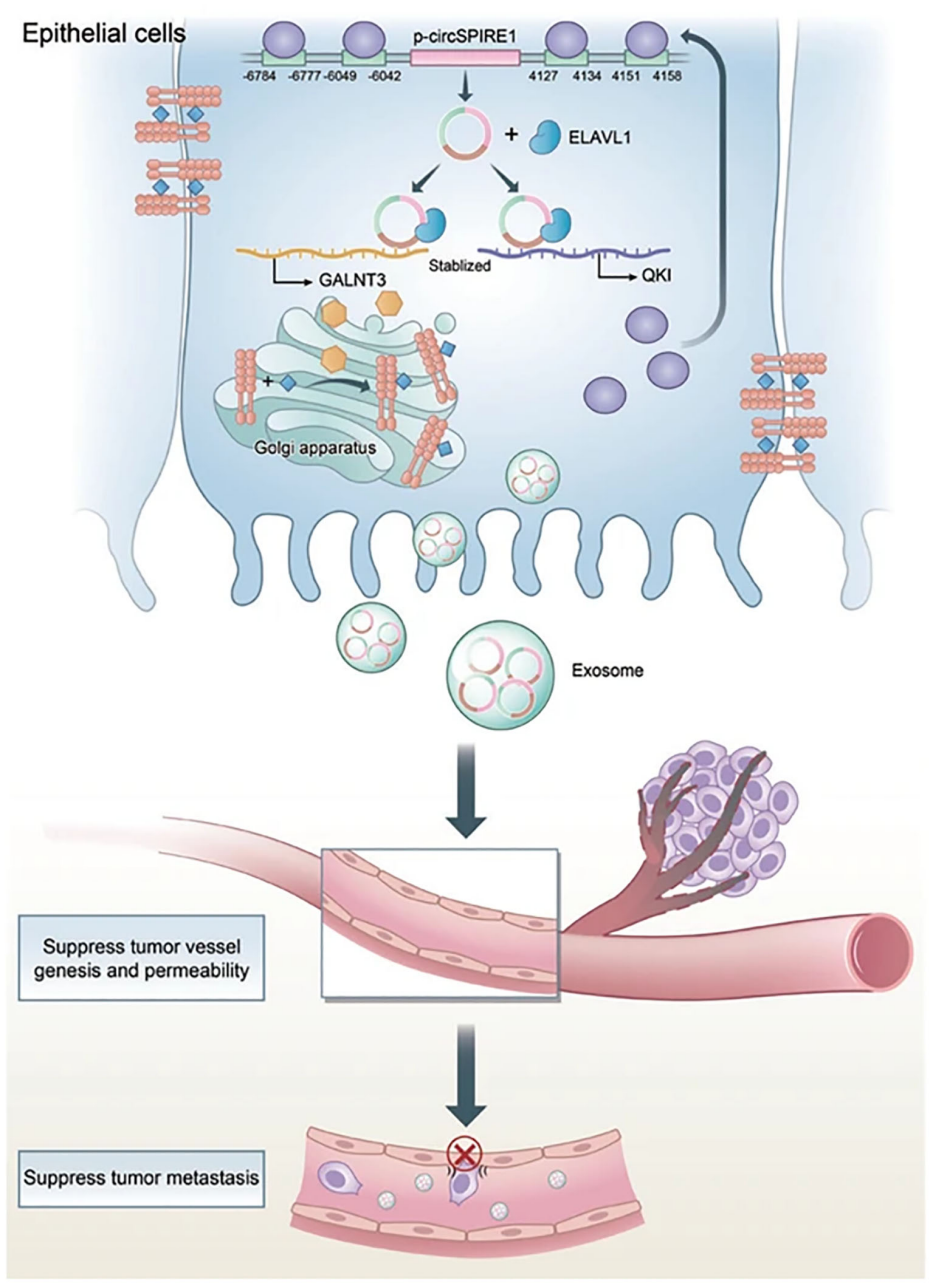
Mechanism of circSPIRE1 during RCC metastasis in tumor microenvironment. Reprinted with permission from Shu G, Lu X, Pan Y, Cen J, Huang K, Zhou M, et al. Exosomal circSPIRE1 mediates glycosylation of E-cadherin to suppress metastasis of renal cell carcinoma. Oncogene. 2023;42(22):1802-20. Copyright Springer Nature.

One of the functions of exosomes is intercellular communication, and therapies could make full use of it to send signals to prevent RCC metastasis. For instance, Yoshino et al. have found that miRNA-1 (miR-1) could suppress RCC growth and invasion. Their experiment has shown that the miR-1 expression would be elevated in the RCC cells if they were treated with exosomes produced by miR-1-transfected cells. They believed that this interesting finding would be a potential treatment for RCC (62). Besides, IL-12-anchored renal cancer cells could produce exosomes expressing renal cell carcinoma-associated antigen G250 and glycolipid-anchored-IL-12 (GPI-IL-12). Surprisingly, exosomes with GPI-IL-12 could significantly promote the proliferation of T cells and later would increase the release of IFN-γ. Besides, stimulating exosomes with GPI-IL-12 could effectively induce antigen-specific cytotoxic T lymphocytes (CTLs), which results in significant cytotoxic effects. All these results have shown that exosomes derived from IL-12-anchored renal cancer cells could express GPI-IL-12 and G250, which may be applied to treating RCC in the future (63). In addition, Brossa and colleagues have conducted experiments about the influence of extracellular vesicles (EVs) produced by human liver stem cells (HLSCs) on renal CSCs. Through systemic administration, EVs from HLSCs could suppress the subcutaneous tumor growth by decreasing the vascularization of the tumor and inducing the apoptosis of tumor cells, which may result from the transportation of antitumor miRNAs. This would also be a possible method to treat RCC in the future (64). Moreover, another study found that exosomal circSPIRE1 could suppress RCC metastasis. It could upregulate polypeptide N-acetylgalactosaminyltransferase 3 (GALNT3) and KH domain RNA binding protein (QKI) expression. GALNT3 could promote glycosylation and cytomembrane localization of E-cadherin and QKI could form a positive feedback loop to enhance circSPIRE1 expression. Additionally, exosomal circSPIRE1 could also suppress angiogenesis and vessel permeability (65) (**Figure 4**).

### 5.2 Suppressing the formation of exosomes could reduce their bad effects

**Figure 5:**
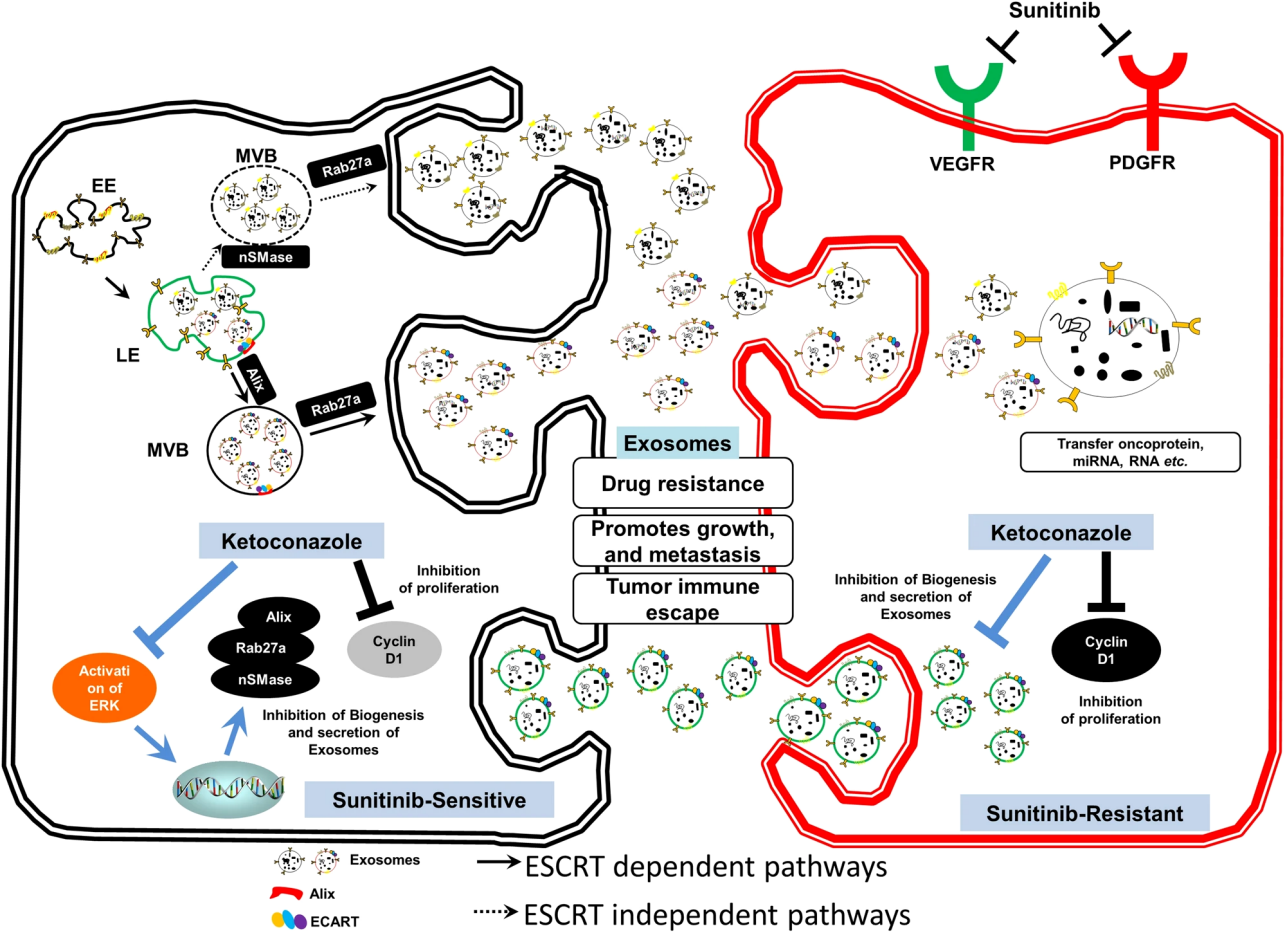
Exosome-mediated transfer of therapeutic resistance in the RCC microenvironment by the clinically approved KTZ. Reprinted with permission from Greenberg JW, Kim H, Moustafa AA, Datta A, Barata PC, Boulares AH, et al. Repurposing ketoconazole as an exosome directed adjunct to sunitinib in treating renal cell carcinoma. Scientific reports. 2021;11(1):10200. Copyright Springer Nature.

Exosomes could promote RCC proliferation, invasion, metastasis, and drug resistance through intercellular communications while suppressing the formation of exosomes could reduce tumor progression. Greenberg et al. have explored that Ketoconazole (KTZ), a kind of anti-fungal medicine, could suppress the biogenesis and secretion of exosomes, which would be an effective medicine for RCC treatment because it could inhibit the RCC metastasis induced by exosomes (66) (**Figure 5**). The same research team also claimed that sunitinib resistance (SR) of RCC is due to existing SR exosomes, while the use of tipifarnib decreases the expression of PD-L1 protein and SR exosome production and secretion (67). Besides, Wang et al. have proposed a transformable dual-inhibition system (TDS) that could reduce the transportation of exosomes produced by CSCs, which could inhibit the delivery of miR-19b. This could increase the expression of PTEN and suppress the RCC metastasis mediated by CSCs (68).

## 6 Discussion and future perspectives

**Figure 6:**
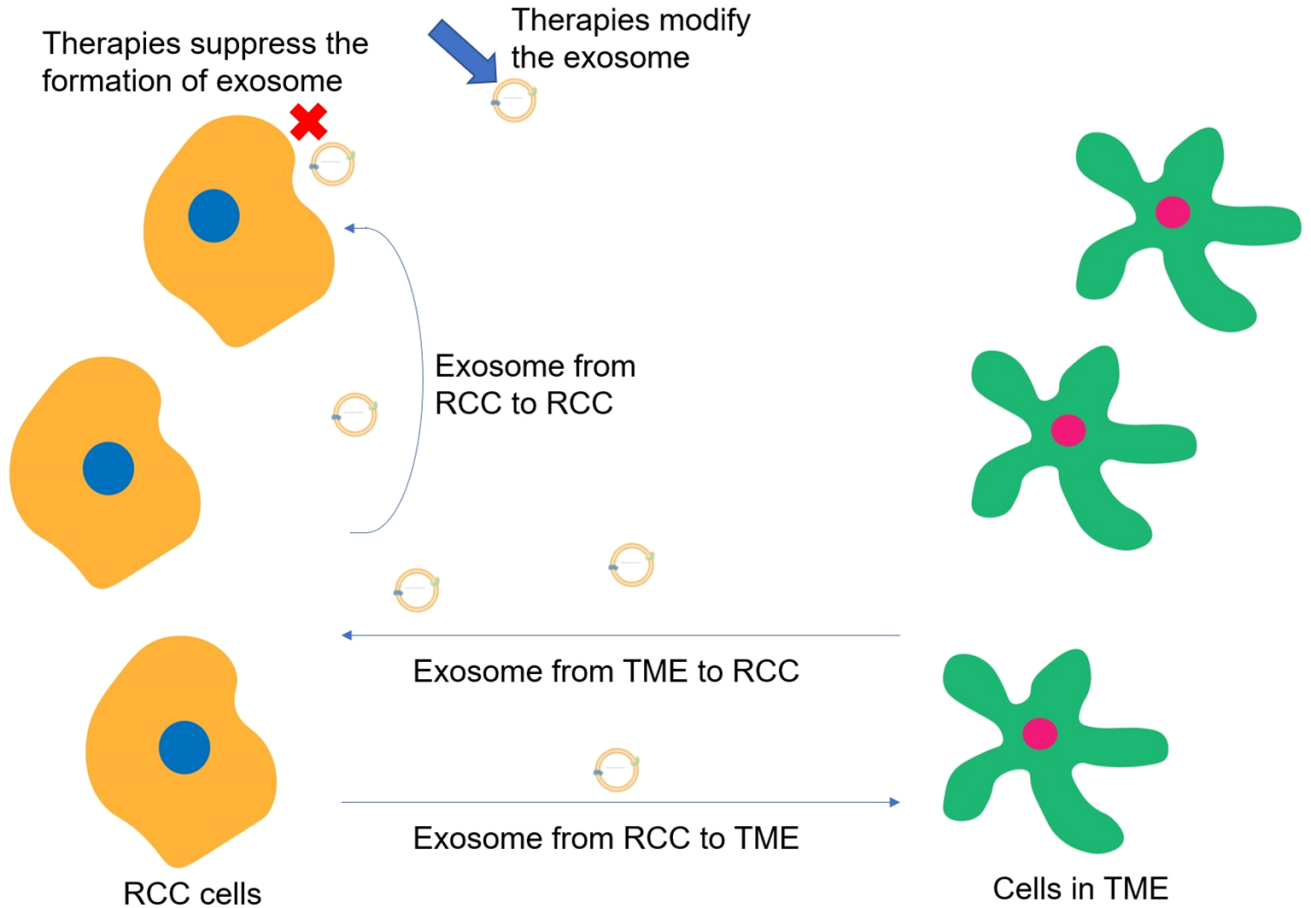
Major roles of exosomes in RCC progression and ways for potential treatments.

The major role of exosomes in RCC metastasis could be divided into regulating TME through the transportation of exosomes from RCC cells to other cells, inducing the proliferation and metastasis of RCC cells via transporting exosomes from TME to RCC cells, and promoting tumor metastasis by the transportation of exosomes from RCC cells to RCC cells. When the immune-related cells in the TME are recipient cells of exosome transportation, the main function of exosome trafficking is to suppress the immune system. However, when RCC cells are the recipient cells of exosome transportation, the main function of exosome trafficking is to promote RCC proliferation and metastasis through signaling pathways. Moreover, though only limited studies have focused on the utilization of exosomes in treating RCC, the main function of exosomes in targeted therapy could be also categorized into two types, which are utilizing the intercellular communications of exosomes to inhibit RCC metastasis and suppressing the exosome formation to reduce their bad effects (**Figure 6**). On the one hand, exosomes could be genetically engineered or chemically modified, including the expression of specific proteins or ligands on their surfaces to enhance the selective binding ability to RCC cells and target cells within the TME, or could be loaded with therapeutic molecules, such as miRNAs, lncRNAs, to regulate gene expression or signaling pathways within the target cells to activate the immune response and cancer inhibition-related signaling pathways within the TME. On the other hand, cutting off exosome-associated intercellular communication and preventing the activation of pro-cancer pathways through exosome-neutralizing antibodies or competitive antibodies, reducing the likelihood of immunosuppression and immune cell depletion in TME. The key molecules VHL and mTOR, as well as the critical pathways VHL-HIF and PI3K/Akt/mTOR, may be the prime targets in kidney cancer therapy (69). These involved cells, molecules, and pathways may become targets for the exosome-based treatment of renal cell carcinoma.

Up to now, there have been four clinical trials involving exosomes and renal cancer, and the main focus of these studies has been on using exosomes to evaluate the efficacy of renal cancer treatments and as early biomarkers for renal cancer. NCT02439008, NCT02071719, and NCT05705583 are three studies that assess the effectiveness of different treatment modalities for renal cancer through exosomes. NCT04053855 is a study that aims to detect tumor exosomes in urine to provide a new liquid biopsy tool for the early diagnosis of clear cell renal cell carcinoma (https://clinicaltrials.gov). It is evident that the application of exosomes in targeted therapies for renal cancer requires further exploration of their potential.

Besides, according to the examples listed above, exosomes mostly function as the carriers of RNAs and proteins, while non-coding RNAs especially microRNAs consist of the majority of exosome cargos. Different non-coding RNAs have different functions and may suggest different stages of tumor progression, so the upregulation of specific non-coding RNAs in the body fluid could be detected and may be promising markers for the early diagnosis of localized and metastatic RCC separately (70). In addition, drug resistance is a big problem in RCC treatment, while some non-coding RNAs have been proven to play an important role in RCC proliferation and sunitinib resistance (71). Preventing the formation of harmful exosomes, targeting the conveyance of harmful exosomes, and inhibiting the harmful exosomes from binding to recipient cells may work as useful tools to prevent RCC proliferation and sunitinib resistance, resulting in a better prognosis for RCC treatments. Perhaps in the near future, targeted therapy targeting these harmful exosomes or targeting some molecules carried by exosomes will become a new approach in cancer treatment. Nonetheless, exosomes are also a double-edged sword, which means that not all exosomes are harmful to the human body and promote tumor proliferation and migration. Some beneficial exosomes trafficking certain molecules would also be helpful in activating immune response to suppress tumor progression (72). It is also worth trying to modify the exosomes to obtain beneficial functions or trying to let exosomes carry therapeutic drugs may be a problem solver for the current situation, which would also be a breakthrough point in exosome-based targeted therapy. Exosomes possess significant advantages as natural carriers in targeted therapy. First, they can effectively avoid phagocytosis by immune cells and freely cross the vessel wall and extracellular matrix, ensuring the wide distribution and stability of exosomes in biological fluids. Second, compared with synthetic nanoparticles, exosomes are derived from human cells and have better biocompatibility and lower immunogenicity. Exosomes can enter target cells through receptor-mediated endocytosis, which optimizes the endocytosis process and promotes the effective internalization of drugs. What’s more, exosomes have a natural targeting ability to penetrate biological barriers, such as the blood-brain barrier, making them highly promising carriers for targeted drugs (11). However, there are still a number of issues that need to be addressed before this goal can be realized. It is necessary to confirm that these molecules can inhibit tumor progression, and then there should be appropriate ways to link them with the transportation of exosomes in order to be applied to the researches on the treatment of renal cell carcinoma. Besides, large-scale production of exosomes is limited by their short circulating lifespan, inaccurate targeting ability, and inappropriate controls (73).

## 7 Conclusions

In a nutshell, metastatic RCC is a disease that still lacks effective treatments though targeted therapy and immunotherapy have brought hope to doctors and patients. Since exosomes were first discovered, the discussion about their connection with cancers has never stopped. Exosomes are important carriers for intercellular transportation and communications, which could deliver small molecules from one cell to another. Studies have found the secrets behind RCC metastasis related to exosomes, which are the cell transportation from RCC to TME, from TME to RCC, and from RCC to RCC. Based on these findings, many doctors have proposed that their findings about the mechanism of RCC metastasis would have a promising future in treating metastatic RCC. However, till now, limited papers have reported their research advance in therapy targeting exosomes, and most of these researches were conducted on the model. Nonetheless, as for some other diseases, utilizing exosomes for carrying certain medicines and inhibiting the formation of exosomes have already been the pilot therapies. Probably, in the near future, exosomes will be a promising target for renal cell carcinoma treatment.
